# Water-Soluble Vitamins: Hypo- and Hypervitaminosis in Pediatric Population

**DOI:** 10.3390/pharmaceutics17010118

**Published:** 2025-01-16

**Authors:** Roberto Paparella, Fabiola Panvino, Lucia Leonardi, Ida Pucarelli, Michela Menghi, Ginevra Micangeli, Francesca Tarani, Marcello Niceta, Debora Rasio, Rouzha Pancheva, Marco Fiore, Luigi Tarani

**Affiliations:** 1 Department of Maternal Infantile and Urological Sciences, Sapienza University of Rome, 00161 Rome, Italyluigi.tarani@uniroma1.it (L.T.); 2 Department of Human Neuroscience, Section of Child and Adolescent Neuropsychiatry, Sapienza University of Rome, 00161 Rome, Italy; 3 Department of Molecular Genetics and Functional Genomics, Ospedale Pediatrico Bambino Gesù, IRCCS, 00100 Rome, Italy; 4 Department of Clinical and Molecular Medicine, Sapienza University of Rome, 00161 Rome, Italy; 5 Department of Hygiene and Epidemiology, Faculty of Public Health, Medical University Prof Dr Paraskev Stoyanov, 9002 Varna, Bulgaria; 6 Department of Sensory Organs, Institute of Biochemistry and Cell Biology (IBBC-CNR), Sapienza University of Rome, 00161 Rome, Italy

**Keywords:** hypovitaminosis, hypervitaminosis, water-soluble vitamins, pediatric nutrition, vitamin sources

## Abstract

Background/Objectives: Water-soluble vitamins, comprising the B-complex vitamins and vitamin C, are essential for normal growth, cellular metabolism, and immune function in pediatric populations. Due to limited storage in the body, these vitamins require consistent intake to prevent deficiencies. Pediatric populations, particularly infants and young children, face a heightened risk of both deficiency and, in rare cases, toxicity due to varying dietary intake and increased developmental needs. This review explores the clinical importance of water-soluble vitamins, focusing on hypo- and hypervitaminosis in children. Methods: A narrative review of the recent literature on the sources, recommended intakes, deficiency symptoms, and potential toxicities associated with each water-soluble vitamin was conducted. Results: Deficiencies in water-soluble vitamins can lead to diverse clinical outcomes, such as neurological, hematological, and immune-related symptoms, depending on the specific vitamin involved. Pediatric populations with increased nutritional needs, such as those experiencing rapid growth or with malabsorption conditions, are particularly vulnerable to vitamin insufficiencies. Conversely, although uncommon, excessive intake of certain water-soluble vitamins may cause mild toxicity, primarily gastrointestinal or neurological. Conclusions: Monitoring water-soluble vitamin levels and providing tailored nutritional support are critical to prevent the adverse effects of hypo- and hypervitaminosis in children. Further research is needed to refine pediatric nutritional guidelines and address the specific needs of young patients, supporting optimal health outcomes.

## 1. Introduction

Water-soluble vitamins, comprising the B-complex group and vitamin C, are essential micronutrients crucial for various metabolic processes, including energy production, neurological function, and immune responses [1,2,3]. Unlike fat-soluble vitamins, water-soluble vitamins are not significantly stored in the body and are more susceptible to depletion, particularly during periods of rapid growth or inadequate dietary intake [4,5]. Pediatric patients, especially those in developmental phases with increased metabolic demands, face unique risks for both deficiencies (hypovitaminosis) [6] and, less commonly, toxicities (hypervitaminosis) [7]. This review examines the clinical features of hypo- and hypervitaminosis of water-soluble vitamins in pediatric populations, drawing on current evidence to clarify the sources, recommended dietary intakes (RDIs), and clinical manifestations associated with these vitamins.

In addition to the RDIs provided by the Institute of Medicine (IOM) and those used in the current review [8,9], it is important to consider that guidelines for water-soluble vitamins can vary significantly between countries and regions. This variability can arise due to differences in dietary habits, lifestyle factors, and health conditions specific to each population. For example, the European Food Safety Authority and the World Health Organization provide alternative recommendations that may differ slightly from those set by the IOM, particularly for certain vitamins like B12 and C, depending on regional dietary patterns [10,11]. In some Asian countries, where the diet may be higher in rice and lower in certain fruits and vegetables, recommendations for vitamins such as B1 (thiamine) and C may be adjusted to reflect regional nutritional gaps [12]. Therefore, when considering water-soluble vitamin recommendations, it is essential to acknowledge these global differences in dietary intake and health needs to ensure the broad applicability of these guidelines.

Furthermore, while RDIs provide valuable guidance on daily vitamin intake, plasma levels of water-soluble vitamins offer a more accurate reflection of nutritional status and potential deficiency or adequacy [13], as they reflect the body’s reserves and are not influenced by short-term fluctuations in dietary intake. Measuring these levels (if available), along with other biomarkers (like methylmalonic acid or homocysteine, when needed), enables a precise evaluation of vitamin status, particularly in high-risk populations. This approach enhances the understanding and management of vitamin imbalances in clinical and public health contexts.

## 2. Materials and Methods

In October 2024, a comprehensive literature search was conducted by three authors to identify relevant manuscripts across multiple databases, including PubMed, Scopus, and Web of Science (WOS), to support this narrative review. Articles were selected using keywords such as “hypovitaminosis”, “hypervitaminosis”, “water-soluble vitamins”, “pediatric nutrition”, and “vitamin sources”, with no restriction on publication year. Inclusion criteria were as follows: (1) English-language articles, (2) original studies on hypovitaminosis, (3) original studies on hypervitaminosis, (4) research focusing on water-soluble vitamins, and (5) studies addressing pediatric nutrition. Letters, editorials, and case reports were included where appropriate. Studies meeting these criteria were further analyzed, and relevant data were extracted from each paper.

## 3. Vitamin B1 (Thiamine)

### 3.1. Sources of Vitamin B1

Thiamine, or vitamin B1, is an essential water-soluble vitamin primarily obtained from whole grains, legumes, nuts, and seeds. Pork and liver also provide rich sources of thiamine, along with fortified cereals and breads commonly available in many countries (Table 1) [8]. Thiamine-fortified formula or foods are essential for infants, especially in populations with a high prevalence of polished rice-based diets, which lack this nutrient. Thiamine is sensitive to heat and prolonged cooking, which can lead to nutrient loss, so steaming or minimal heat exposure is often recommended to preserve its content [14].

### 3.2. RDIs and Physiological Functions

Thiamine is essential for cellular function, acting as a coenzyme in the metabolism of carbohydrates, fats, and proteins. It supports mitochondrial energy production, fatty acid oxidation, and protein synthesis. Additionally, thiamine is vital for proper central and peripheral nervous system function, including neurotransmitter production [15]. RDIs vary with age, starting from 0.2 mg/day in infants to 1.2 mg/day in adolescents (Table 2) [8]. Sufficient intake is particularly important in children undergoing rapid growth or increased metabolic demand.

### 3.3. Hypovitaminosis B1

Thiamine deficiency in children can lead to conditions like beriberi, characterized by symptoms including irritability, muscle weakness, and cardiac issues such as tachycardia and cardiomegaly. The presence of neurological involvement is referred to as “dry beriberi”, characterized by polyneuritis and symmetrical muscle wasting. Severe deficiency may result in Wernicke’s encephalopathy, though rare in children, which is denoted by the triad of confusion, ophthalmoplegia, and ataxia. “Wet beriberi” refers to high-output cardiac failure, with edema and dyspnea on exertion [16]. Risk factors include malnutrition, malabsorption syndromes, alcohol dependency, and excessive reliance on thiamine-poor foods [17].

### 3.4. Hypervitaminosis B1

Due to its water solubility, excessive intake of thiamine is rare, with minimal risk of toxicity. High doses, generally from supplements, may result in mild side effects, such as headaches and irritability, but serious adverse effects are uncommon due to efficient renal excretion. The available evidence from clinical studies, along with the long history of therapeutic use, suggests that current intake levels of vitamin B1 from all sources do not pose a health risk to the general population [18].

## 4. Vitamin B2 (Riboflavin)

### 4.1. Sources of Vitamin B2

Riboflavin, or vitamin B2, is widely found in animal and plant-based foods, including milk, eggs, lean meats, green leafy vegetables, and fortified cereals (Table 1) [8]. Its availability from dairy products makes riboflavin intake accessible to children with balanced diets. Riboflavin is light-sensitive, so milk and other foods are best stored away from direct sunlight to maintain potency [19].

### 4.2. RDIs and Physiological Functions

Riboflavin is important for energy production and the functioning of other B vitamins. It is a precursor of the essential coenzymes flavin mononucleotide and flavin adenine dinucleotide, both recognized by antioxidant properties and involved in oxidation-reduction reactions, with a great impact on energy metabolism [20]. They are also required for the biosynthesis of niacin and the conversion of pyridoxine into its active form [21]. Intake recommendations range from 0.3 mg/day in infants to 1.3 mg/day in adolescents (Table 2) [8]. Given its role in growth and development, riboflavin requirements increase with activity levels and age [22].

### 4.3. Hypovitaminosis B2

Severe riboflavin deficiency, though uncommon and mainly found in low-income regions, presents with cheilosis, angular stomatitis, glossitis, seborrheic dermatitis, and anemia due to erythroid hypoplasia [23]. Children with limited diets, malabsorption issues, or low dairy intake are particularly at risk [22]. Moreover, ultraviolet light can degrade riboflavin, so it is always stored in opaque containers to protect it [24].

### 4.4. Hypervitaminosis B2

Toxicity from riboflavin is rare, as excess amounts are easily excreted in urine. Even at high supplemental doses, adverse effects are minimal, with no well-documented cases of riboflavin toxicity in children [25].

## 5. Vitamin B3 (Niacin)

### 5.1. Sources of Vitamin B3

Niacin, available in two forms—nicotinic acid and nicotinamide—is found in meat, fish, poultry, and whole grains. Fortified grains and cereals contribute significantly to dietary intake in pediatric populations (Table 1) [8]. Some niacin can also be synthesized endogenously from tryptophan, particularly when protein intake is adequate [26].

### 5.2. RDIs and Physiological Functions

Niacin is vital for DNA repair, energy production, and antioxidant protection. The active form is either nicotinamide adenine dinucleotide or nicotinamide adenine dinucleotide phosphate, key components of intermediary metabolism [27]. Recommendations increase with age, ranging from 2 mg/day for infants to 16 mg/day for adolescents (Table 2) [8].

### 5.3. Hypovitaminosis B3

Niacin deficiency can cause pellagra, and nutritional insufficiency was once its most common cause worldwide [28]. However, in developed countries, primary nutritional deficiency rarely causes pellagra anymore, as it has nearly been eradicated. Instead, hypovitaminosis B3 now typically arises as a result of chronic alcoholism (not common in pediatric age), malabsorption syndromes, certain medications, and anorexia nervosa [29]. Changes in the skin, gastrointestinal tract, and nervous system are observed in individuals with pellagra. Typical symptoms include dermatitis, diarrhea, and, in severe cases, neurological effects like dementia (the 3-D’s) [30]. Low levels of pyridoxine interfere with tryptophan biosynthesis, which, in turn, might determine niacin deficiency, producing secondary pellagra, indistinguishable from the primary form [31].

### 5.4. Hypervitaminosis B3

Excessive niacin intake, primarily from supplements, can result in flushing, pruritus, and, with very high doses, liver toxicity [32]. Monitoring is recommended in cases where supplementation exceeds dietary intake significantly, particularly in pediatric populations. Moreover, studies in the literature have shown that niacin can be used to treat dyslipidemias in pediatric patients, but the aforementioned adverse events should be monitored, as their occurrence is not uncommon [33].

## 6. Vitamin B5 (Pantothenic Acid)

### 6.1. Sources of Vitamin B5

Pantothenic acid is found in almost all food sources, particularly in meat, poultry, whole grains, and eggs. Avocado, broccoli, and fortified cereals are also rich in vitamin B5 (Table 1) [8], making deficiency uncommon when a balanced diet is consumed, due to its widespread presence in foods [34].

### 6.2. RDIs and Physiological Functions

Vitamin B5 plays a vital role in synthesizing coenzyme A [35], which is essential for fatty acid metabolism and energy production. The structure of CoA acts as a carbonyl-activating agent and an acyl group carrier, aiding in the facilitation of various reactions. It is indeed involved in the synthesis of fatty acids, cholesterol, steroid hormones, amino acids, and neurotransmitters [36]. The RDIs range from 1.7 mg/day in infants to 5 mg/day in adolescents (Table 2) [8].

### 6.3. Hypovitaminosis B5

Though rare, pantothenic acid deficiency can lead to symptoms such as fatigue, irritability, numbness, and muscle cramps, collectively termed “burning feet syndrome”. This condition is more prevalent in populations with severe malnutrition or specific malabsorption syndromes, being more likely to occur in conjunction with multiple nutritional deficiencies [37].

### 6.4. Hypervitaminosis B5

Pantothenic acid toxicity is very rare due to its water solubility, and excess intake is generally well-tolerated [1]. Extremely high doses may result in mild gastrointestinal symptoms [8], but no severe toxicity has been reported in children or adults.

## 7. Vitamin B6 (Pyridoxine)

### 7.1. Sources of Vitamin B6

Pyridoxine is available in several forms and is abundant in foods such as fish, poultry, potatoes, chickpeas, and fortified cereals. Bananas and leafy green vegetables also contain vitamin B6, though in lesser amounts than animal-based sources (Table 1) [8].

### 7.2. RDIs and Physiological Functions

The active biochemical form of pyridoxine is pyridoxal phosphate. As a coenzyme, it is a co-factor in numerous enzymatic reactions, including lipid, amino acid, and carbohydrate metabolism [38]. It is critical in transamination and decarboxylation, the initial steps of porphyrin synthesis. Vitamin B6 plays also a role in neurotransmitter synthesis and immune function [39]. RDIs increase with age, from 0.1 mg/day in infants to 1.3 mg/day in adolescents (Table 2) [8].

### 7.3. Hypovitaminosis B6

Pyridoxine insufficiency can lead to irritability and seizures in children. Symptoms such as depression, confusion, fatigue, and peripheral neuropathy may also be present [40]. Pellagra symptoms can arise due to pyridoxine deficiency, which disrupts the conversion of tryptophan into niacin [31]. Distinct conditions of pyridoxine-dependent seizures may manifest in infancy, responsible for severe epilepsy, resistant to antiepileptic drugs but responsive to pharmacological dosages of vitamin B6 [41]. A deficiency in vitamin B6 may also affect the blood and skin, manifesting as microcytic anemia and seborrheic dermatitis, respectively [42]. Populations at risk include exclusively breast-fed infants older than 6 months, as well as children with malabsorption disorders, prolonged restrictive diets, renal impairment, and autoimmune disorders [42,43]. Additionally, since isoniazid competitively inhibits the action of pyridoxine, routine supplementation is recommended in children and adults with tuberculosis due to the high risk of B6 deficiency [44].

### 7.4. Hypervitaminosis B6

Pyridoxine is unique in that both deficiency and toxicity cause peripheral neuropathy. High doses of vitamin B6 from supplements can lead to peripheral and/or sensory neuropathy, resulting in paresthesia, burning feet, and sensory ataxia [31]. The severity of symptoms is dose-dependent, but neuropathy may not be reversible despite discontinuing pyridoxine. In addition to neuropathy, non-neurologic symptoms include photosensitivity, nausea and heartburn, and painful skin eruptions; these typically resolve after discontinuation of the vitamin [39]. However, toxicity is rare at dietary intake levels and typically only occurs at supplement doses far above the RDIs.

## 8. Vitamin B7 (Biotin)

### 8.1. Sources of Vitamin B7

Biotin is commonly found in foods such as eggs, nuts, seeds, and certain vegetables, including spinach and broccoli. The liver is particularly rich in biotin (Table 1) [8], and many foods are fortified with this vitamin in some regions. Gut bacteria can also produce biotin, which contributes to overall availability [45].

### 8.2. RDIs and Physiological Functions

Biotin functions as a coenzyme in several carboxylation reactions essential for fatty acid synthesis and energy metabolism [46]. Novel roles of biotin have additionally been recognized, particularly regarding its involvement in cellular signaling and epigenetic regulation [47]. Since biotin is a contributor to keratin, it has also become popular as a supplement to improve hair, skin, and nail quality [48]. RDIs range from 5 μg/day in infants to 25 μg/day in adolescents (Table 2) [8].

### 8.3. Hypovitaminosis B7

Because intestinal bacteria can synthesize adequate levels of biotin, deficiency is rare. Although uncommon, it can lead to symptoms like alopecia, dermatitis, and neurological symptoms such as lethargy and developmental delays [49]. Biotin deficiency can arise from various causes, including rare metabolic disorders like holocarboxylase synthetase deficiency and biotinidase deficiency, the latter being an autosomal recessive condition that leads to neurological and dermatological symptoms due to impaired biotin recycling [50]. Gastrointestinal imbalances from broad-spectrum antibiotics or inflammatory bowel disease can also hinder biotin synthesis [51]. Additionally, individuals on parenteral nutrition without biotin supplementation, and those taking certain antiepileptic medications or isotretinoin may experience low biotin levels [52,53,54]. High consumption of raw egg whites, which contain avidin that binds to biotin, can further contribute to deficiency [55].

### 8.4. Hypervitaminosis B7

Biotin toxicity is rare, and no adverse effects have been reported at high intake levels, likely due to efficient renal excretion. However, the use of biotin must be carefully reconsidered, considering that pharmacological levels may impact physiological functions, and a significant increase in dietary biotin concentration has been demonstrated to alter various aspects of the female mouse reproductive system [56]. Moreover, excessive supplementation may interfere with certain laboratory tests, such as thyroid and cardiac markers. In immunoassays utilizing the streptavidin-biotin interaction, the interference may induce both false-positive and false-negative results [57,58].

## 9. Vitamin B9 (Folate)

### 9.1. Sources of Vitamin B9

Folate, naturally occurring in foods such as leafy green vegetables, legumes, seeds, and liver, is also available as folic acid in fortified cereals and breads (Table 1) [8]. Folate is sensitive to heat and can degrade with prolonged cooking, so minimal heat is advised to retain its nutrient value [59].

### 9.2. RDIs and Physiological Functions

Though the expressions “folic acid” and “folate” are commonly utilized interchangeably, folates represent the naturally occurring form, whereas folic acid is the synthetic form employed for food enrichment and nutritional supplements [60]. Folate is essential for one-carbon methyl transfer in various cellular processes, including purines and pyrimidines synthesis to constitute DNA and RNA. It is thus crucial for nucleic acid synthesis, cell division, and amino acid metabolism [61]. Daily requirements range from 65 μg for infants to 400 μg for adolescents (Table 2) [8].

### 9.3. Hypovitaminosis B9

Megaloblastic anemia due to folate deficiency is characterized by impaired DNA synthesis, leading to the production of abnormally large, immature red blood cells (megaloblasts) in the bone marrow. Clinically, children may display symptoms such as fatigue, pallor, and shortness of breath, along with macrocytic red blood cells on a complete blood count [62]. Neurological impairment may also arise due to folate deficiency. Hereditary folate deficiency frequently results in severe anemia and intellectual disability. Additionally, folate deficiency is the most prevalent cause of elevated plasma homocysteine levels [63]. In pregnant women, inadequate folate intake is linked to an increased risk of neural tube defects in infants, highlighting the importance of supplementation in women of childbearing age [64].

### 9.4. Hypervitaminosis B9

Excessive folic acid intake from supplements can mask vitamin B12 deficiency symptoms, potentially delaying diagnosis and treatment; anemia may be resolved, but neuropathy advances unnoticed to a stage where it becomes irreversible, even with vitamin B12 treatment [65]. Additionally, high doses may interfere with some medications, underscoring the importance of adhering to recommended intake levels [65]. Excessive supplementation during gestation might be associated with insulin resistance and greater adiposity in children under five years of age [66]. Proposed mechanisms for the heightened risk of cancer linked to excessive folic acid intake include the promotion of cellular DNA synthesis and replication while diminishing the natural killer cell response to cancerous cells [67,68].

## 10. Vitamin B12 (Cobalamin)

### 10.1. Sources of Vitamin B12

Vitamin B12 is primarily found in animal-derived foods, such as meat, poultry, fish, eggs, and dairy products. Fortified foods and supplements (Table 1) [8] provide an alternative source for vegetarians and vegans, as B12 is not naturally present in plant-based foods [69].

### 10.2. RDIs and Physiological Functions

Vitamin B12 is essential for red blood cell formation, neurological function, and DNA synthesis [70]. Cobalamin has the lowest recommended dietary allowance of all vitamins, as it is highly conserved through enterohepatic circulation, being secreted into bile and then reabsorbed from the intestines into the hepatic portal vein [71]. It forms a complex with an intrinsic factor, a glycoprotein secreted by the gastric parietal cells, required for cobalamin absorption in the terminal ileum [72]. Intake recommendations range from 0.4 μg/day in infants to 2.4 μg/day in adolescents (Table 2) [8].

### 10.3. Hypovitaminosis B12

Vitamin B12 deficiency may result in megaloblastic anemia, neuropathy, and developmental delays. Symptoms in children may include irritability, failure to thrive, and developmental regression. Vitamin B12 deficiency arises from four main causes: (1) Autoimmune, as in pernicious anemia, where anti-intrinsic factor antibodies prevent B12 absorption by blocking intrinsic factor [73]; (2) Malabsorption, seen in cases like gastric bypass surgery or intestinal damage (e.g., Crohn’s disease, celiac disease, or tapeworm Diphyllobothrium latum infection) that impair B12 uptake [72]; (3) Dietary insufficiency, particularly in strict vegans after about three years without animal products [74]; and (4) Toxin exposure, such as nitrous oxide, which can induce neurological symptoms [75].

### 10.4. Hypervitaminosis B12

Vitamin B12 toxicity is rare, with no established upper intake level due to limited evidence of adverse effects. Even at high supplement doses, B12 is generally considered safe due to its water-soluble nature and efficient renal clearance. However, high intakes represent no benefit in subjects without malabsorption and should therefore be avoided [8].

## 11. Vitamin C (Ascorbic Acid)

### 11.1. Sources of Vitamin C

Ascorbic acid is abundant in citrus fruits, tomatoes, potatoes, strawberries, kiwifruit, and green leafy vegetables. It is also added as an antioxidant to various processed foods (Table 1) [9]. Cooking reduces its content, so fresh or lightly cooked sources are recommended [76].

### 11.2. RDIs and Physiological Functions

Vitamin C is an electron donor (reducing agent) essential for its protective role as an antioxidant, as well as for collagen synthesis, immune function, and iron absorption [77,78]. Intake recommendations range from 40 mg/day in infants to 75 mg/day for adolescent males and 65 mg/day for adolescent females (Table 2) [9].

### 11.3. Hypovitaminosis C

Vitamin C deficiency, though rare, results in scurvy, marked by symptoms such as gum bleeding, petechiae, poor wound healing, and fatigue [79]. Primary alterations of infantile scurvy occur in areas of rapid bone growth; typical signs include a pseudoparalysis of the limbs due to subperiosteal hemorrhages, as well as gum swelling and bleeding around erupting teeth [80,81]. Populations with limited fruit and vegetable intake, including infants on unfortified formulas, are at higher risk [81].

### 11.4. Hypervitaminosis C

Though water-soluble, excessive vitamin C intake can lead to gastrointestinal disturbances, including diarrhea and abdominal cramps [82]. In rare cases, high doses may contribute to kidney stone formation in susceptible individuals [83,84,85].

## 12. Diagnostic and Therapeutic Considerations

Pediatric assessment of water-soluble vitamin imbalances requires specific biomarker measurement, including serum levels (Table 3) and functional assays (e.g., complete blood count for vitamin B deficiencies, and plasma ascorbate for vitamin C). While it may be tempting to measure urine or serum levels of water-soluble vitamins, these only indicate current circulating levels, not providing an estimate of stored vitamin levels. Other testing methods include immunoassays, chromatographic techniques, chemical assays, high-pressure liquid chromatography, and capillary electrophoresis, with the choice of method depending on the specific vitamin being assessed [86]. Targeted testing, such as a complete blood count (including measurements of mean corpuscular volume, hematocrit, and hemoglobin) along with testing for methylmalonic acid and homocysteine levels for vitamin B12 and folate deficiency [87], is critical for identifying certain insufficiencies. Understanding these imbalances, their underlying causes, and associated clinical manifestations is essential for effective management and prevention strategies.

### 12.1. Hypovitaminosis

Deficiency of water-soluble vitamins, including the B-complex group and vitamin C, can lead to diverse clinical manifestations, especially in children with increased nutritional needs or inadequate intake. These vitamins are vital for energy metabolism, immune function, neurodevelopment, and growth. Unlike fat-soluble vitamins, they are not stored in significant amounts, necessitating regular dietary intake. Diagnosis involves clinical evaluation and laboratory tests, including plasma or serum levels and functional biomarkers like homocysteine. Addressing underlying causes, dietary interventions, and targeted supplementation based on severity and age-specific needs are essential for treatment. Prevention strategies should focus on dietary diversity and cautious supplementation, especially for children at high risk of deficiency, which might be a presenting feature in alcohol use disorder, malnutrition, and malabsorption conditions like short-bowel syndrome [88,89].

### 12.2. Hypervitaminosis

Hypervitaminosis of water-soluble vitamins is uncommon but can occur due to excessive supplementation or impaired renal clearance [90]. These vitamins are typically excreted in urine, which minimizes the risk of toxicity [91]. However, high-dose supplementation can overwhelm renal clearance, especially for vitamins like B6 and C. Reduced renal function significantly increases the risk of hypervitaminosis, as impaired kidneys cannot adequately excrete excess vitamins [92]. Although most water-soluble vitamins are not stored in the body, exceptions like vitamin B12, which is stored in the liver, can accumulate if intake is excessive. The clinical manifestations of hypervitaminosis vary by vitamin [7]. Infants, pregnant individuals, and those with metabolic disorders are particularly vulnerable to these toxic effects due to altered metabolic and excretory capacities. Diagnosis relies on clinical presentation and laboratory assessments, including elevated plasma vitamin levels and biomarkers like methylmalonic acid. Factors influencing hypervitaminosis include supplement bioavailability, dietary patterns (e.g., excessive intake of fortified foods), and individual genetic predispositions. Sustained-release or liposomal formulations of vitamins can also increase absorption, raising the potential for toxicity. With the increasing use of high-dose supplements, awareness of these mechanisms and risks is essential in pediatric practice and public health [93].

### 12.3. At-Risk Pediatric Populations

Children with specific conditions, such as malabsorption syndromes or chronic illnesses, have nutritional needs that differ significantly from those of healthy children, who typically meet their requirements through a balanced diet. For example, malabsorption syndromes such as celiac disease or inflammatory bowel disease may lead to impaired nutrient absorption and deficiencies in water-soluble vitamins like B12, folate, and vitamin C [94]. This necessitates close monitoring of serum levels and tailored supplementation to address these deficits. Similarly, children with chronic conditions like cystic fibrosis or chronic kidney disease often require higher intake levels of specific vitamins due to altered metabolic demands or losses through excretion [95,96]. In contrast, over-supplementation risks are heightened in these populations due to potential changes in vitamin metabolism or excretion, such as reduced renal clearance in chronic kidney disease [92]. This makes regular assessment of plasma vitamin levels critical to avoid toxicity.

Moreover, the presence of comorbidities in these children, such as reduced appetite or dietary restrictions, can further complicate achieving adequate nutrient intake. However, even in healthy children, certain subpopulations—such as those with limited dietary diversity, food allergies, or socio-economic constraints—may benefit from targeted nutritional support [97,98]. A multidisciplinary approach, involving pediatricians, dietitians, and specialists, is essential to develop individualized dietary and supplementation plans.

## 13. Conclusions

Water-soluble vitamins are critical to pediatric growth and health, with distinct deficiency and toxicity profiles. Early detection and intervention can prevent serious health outcomes, particularly in at-risk pediatric populations. To better understand age-related differences in the frequency of hypo- and hypervitaminosis, future studies should collect population-specific data stratified by age, sex, and clinical context. Such research could clarify how factors like dietary habits, absorption, and metabolic demand influence vitamin deficiencies or excesses at different growth stages, improving age-specific reference ranges and public health strategies. Future research should focus on the broader roles of these vitamins beyond classical deficiency syndromes, such as their impact on neurodevelopment, immune function, and the modulation of gut microbiota. Promising areas of research include the role of genetic and epigenetic factors in vitamin metabolism, the interaction between water-soluble vitamins and chronic diseases, and the development of innovative diagnostic tools for early identification of imbalances. Additionally, studies addressing age-specific prevalence, regional and socioeconomic disparities, and the formulation of precise, evidence-based guidelines for safe and effective supplementation across pediatric age groups are essential to optimize clinical care.

## Figures and Tables

**Table 1:** Main dietary sources of water-soluble vitamins in pediatric nutrition. This table summarizes the primary sources of water-soluble vitamins essential for pediatric health.

Vitamin	Main Sources
B1 (Thiamine)	Whole grains, legumes, pork, nuts, seeds, and fortified cereals
B2 (Riboflavin)	Dairy products (milk, yogurt), eggs, lean meats, green leafy vegetables, and fortified cereals
B3 (Niacin)	Meat, fish, poultry, fortified grains, nuts, seeds, and legumes
B5 (Pantothenic acid)	Meat (chicken, beef), whole grains, potatoes, avocados, eggs, and mushrooms
B6 (Pyridoxine)	Poultry, fish, potatoes, chickpeas, bananas, and fortified cereals
B7 (Biotin)	Eggs (yolk), nuts (almonds, peanuts), seeds, legumes, whole grains, and sweet potatoes
B9 (Folate)	Leafy green vegetables (spinach, kale), legumes (beans, lentils), citrus fruits, fortified cereals, and asparagus
B12 (Cobalamin)	Animal products (meat, fish, dairy, eggs), fortified cereals and plant-based milk for vegans
C (Ascorbic acid)	Citrus fruits (oranges, lemons), strawberries, bell peppers, broccoli, kiwi, tomatoes, and green leafy vegetables

**Table 2:** Recommended dietary intake (RDI) and upper tolerable level (UL) for water-soluble vitamins in pediatric populations. This table summarizes the RDIs and ULs for children and adolescents, expressed in milligrams (mg)/day for vitamins B1, B2, B3, B5, B6, and C, and micrograms (μg)/day for vitamins B7, B9, and B12. The RDIs of water-soluble vitamins vary based on age, sex, and individual health status. The RDI is expressed as recommended dietary allowance (RDA), that is the level of dietary intake sufficient to meet the daily nutrient requirements of 97% of the individuals in a specific life stage group. AI = adequate intake (an approximation of the average nutrient intake that sustains a defined nutritional state, based on observed or experimentally determined values in a defined population; it is used when there is insufficient data to determine the RDI for a given nutrient); ND = not determined (data were insufficient to set a UL).

Age Group	B1 (mg/day)		B2 (mg/day)		B3 (mg/day)		B5 (mg/day)		B6 (mg/day)		B7 (μg/day)		B9 (μg/day)		B12 (μg/day)		C (mg/day)	
	RDA	UL	RDA	UL	RDA	UL	RDA (AI)	UL	RDA	UL	RDA (AI)	UL	RDA	UL	RDA	UL	RDA	UL
0–6 months	0.2 (AI)	ND	0.3 (AI)	ND	2 (AI)	ND	1.7	ND	0.1 (AI)	ND	5	ND	65 (AI)	ND	0.4 (AI)	ND	40 (AI)	ND
7–12 months	0.3 (AI)	ND	0.4 (AI)	ND	4 (AI)	ND	1.8	ND	0.3 (AI)	ND	6	ND	80 (AI)	ND	0.5 (AI)	ND	50 (AI)	ND
1–3 years	0.5	ND	0.5	ND	6	10	2	ND	0.5	30	8	ND	150	300	0.9	ND	15	400
4–8 years	0.6	ND	0.6	ND	8	15	3	ND	0.6	40	12	ND	200	400	1.2	ND	25	650
9–13 years	0.9	ND	0.9	ND	12	20	4	ND	1.0	60	20	ND	300	600	1.8	ND	45	1200
14–18 years	1.2 (male), 1.0 (female)	ND	1.3 (male), 1.0 (female)	ND	16 (male), 14 (female)	30	5	ND	1.3 (male), 1.2 (female)	80	25	ND	400	800	2.4	ND	75 (male), 65 (female)	1800

**Table 3:** Reference ranges and methods of assessment for water-soluble vitamins. This table provides an overview of the reference ranges for plasma levels, primary and alternative measurement methods, and relevant clinical considerations for water-soluble vitamins. Deficiency and adequacy thresholds are outlined based on clinical guidelines and the available literature. Plasma levels should be interpreted in conjunction with dietary intake, clinical symptoms, and risk factors, as systemic conditions (e.g., inflammation or hypoalbuminemia) may affect their accuracy. Functional biomarkers (e.g., transketolase activity for B1, erythrocyte glutathione reductase for B2, and methylmalonic acid for B12) can supplement plasma measurements to better evaluate specific deficiencies. N/A = not available.

Vitamin	Reference Range	Plasma Levels	Primary Method of Measurement	Alternative Methods	Considerations
B1 (Thiamine)	70–180 nmol/L (3.0–7.7 μg/dL)	Variable, may be reduced in inflammation	Blood thiamine concentration (direct)	Erythrocyte transketolase activity, urinary thiamine excretion	Maybe falsely reduced in systemic inflammation; hypoalbuminemia affects the interpretation
B2 (Riboflavin)	Activity coefficient > 1.4 indicates insufficiency	Reflects recent intake	Erythrocyte glutathione reductase assay	Urinary riboflavin excretion	Urinary levels reflect dietary intake but not individual deficiency
B3 (Niacin)	Not standardized	N/A	Urinary N-methyl nicotinamide	Erythrocyte NAD: NADP ratio	Tests not widely available; high urinary N-methyl nicotinamide indicates adequate status
B5 (Pantothenic acid)	Urinary excretion > 1 mg/day indicates sufficiency	N/A	Urinary excretion	Blood/plasma/erythrocyte levels (unreliable)	Urinary excretion correlates with dietary intake; deficiency is rare
B6 (Pyridoxine)	PLP > 30 nmol/L (>7.4 ng/mL) sufficient	Marginal: 20–30 nmol/L	Plasma pyridoxal-5-phosphate (PLP)	Erythrocyte transaminase activity, urinary 4-pyridoxic acid, xanthurenic acid excretion	Elevated xanthurenic acid post-tryptophan load suggests deficiency
B7 (Biotin)	Urinary excretion: 75–195 μmol/day	N/A	Urinary biotin excretion	Serum biotin (less sensitive)	Urinary excretion is preferred; serum levels may not reflect intake or sufficiency
B9 (Folate)	1.8–9 ng/mL (4.1–20.4 nmol/L)	N/A	Serum folate	Red blood cell folate	Serum levels fluctuate with diet; red blood cell folate provides a longer-term indicator
B12 (Cobalamin)	200–800 pg/mL (147.5–589.8 pmol/L)	Suboptimal: <400 pg/mL, Deficient: <200 pg/mL	Serum cobalamin	Holotranscobalamin, methylmalonic acid, homocysteine	Gastric bypass patients at higher risk of deficiency; neuropsychiatric symptoms possible even with normal hematologic findings
C (Ascorbic acid)	Plasma: 23–114 µmol/L (0.4–2.0 mg/dL)	Correlation with intake	Plasma and leukocyte vitamin C levels	High-performance liquid chromatography	No reliable functional tests; reference ranges vary by laboratory

## Data Availability

Not applicable.
